# Drivers of COVID-19 Vaccination among Eligible Adults in Abuja, Nigeria: A Mixed-Methods Study Using the WHO Behavioral and Social Drivers of Vaccination Framework

**DOI:** 10.3390/vaccines12101128

**Published:** 2024-10-01

**Authors:** Chizoba B. Wonodi, Ikechukwu A. Okpe, Pius U. Angioha, Affiong S. Ebong, Janet B. Adegbola, Abdulrasheed A. Abdulraheem, Nwamaka Ezeanya, Adewumi A. Adetola, Oluwatosin I. Arogundade, Goodness I. Hadley, Joseph A. Olisa

**Affiliations:** 1 Department of International Health, International Vaccine Access Center, Johns Hopkins Bloomberg School of Public Health Baltimore, Baltimore, MD 21231, USA; 2 Direct Consulting and Logistics, Maitama, Abuja 904101, Nigeria; okpeamos007@gmail.com (I.A.O.);; 3 Women Advocates for Vaccine Access, Maitama, Abuja 904101, Nigeria

**Keywords:** socio-behavioral drivers, BeSD framework, COVID-19 vaccination, adult vaccination

## Abstract

Despite the availability of COVID-19 vaccines, Nigeria still faces significant COVID-19 vaccine hesitancy, with only 60.7% of the eligible population fully vaccinated as of 20 March 2023. Our study, part of a community-based effort to improve knowledge and uptake of the COVID-19 vaccine in the Gwagwalada Area Council of Abuja, the Federal Capital Territory (FCT) of Nigeria, utilized the WHO’s Behavioral and Social Drivers (BeSDs)-of-vaccination framework to examine the drivers of COVID-19 vaccination among eligible adults. This was a mixed-method study with focus group discussions (FGDs) and in-depth interviews (IDIs) involving 40 purposively sampled participants. We triangulate qualitative findings with data from a household survey of 1512 eligible adults identified using a two-stage systematic cluster sampling approach. All data were collected from the 1–18 November 2022. The household survey showed 46% COVID-19 vaccine uptake, with Pearson chi-square and Fisher’s exact test showing significant associations between vaccine uptake and gender, religion, and education. Multivariate logistic regression showed that confidence in vaccine safety, knowing vaccination sites and family/friends’ endorsement of COVID-19 vaccination were the strongest items associated with vaccine uptake in the thinking-and-feeling, practical-issue, and social-process domains, respectively. Multiple items measuring these domains aligned with BeSD priority question, demonstrating the robustness of the pared-down framework. Qualitative data corroborated these findings. To address vaccine hesitancy and increase uptake, community-driven approaches to improve trust in vaccine safety and benefits and promote positive vaccination norms are needed. In addition, service delivery strategies to make vaccination services easily accessible and identifiable should be developed and tailored to community needs.

## 1. Introduction

It has been more than four years since the first COVID-19 case was reported and more than three years since the first vaccine was administered. Based on the availability of the different approved COVID-19 vaccines and coverage targets set by the country, Nigeria should have vaccinated a large percentage of its adult population, individuals 18 years and older, who were designated the eligible population. However, according to the National Primary Health Care Development Agency (NPHCDA), as of 20 March 2023, only 60.7% of Nigeria’s eligible population had received the full dose of the COVID-19 vaccine. This figure is less than the NPHCDA’s target of 70.3% by December 2022. The literature has reported reasons for the high hesitancy among Nigeria’s population. Some of the reasons include a lack of trust in the government’s intention [1,2], issues with vaccine safety and efficacy [3,4], issues with adverse event following immunization (AEFI) [5], conspiracy theories surrounding COVID-19 and the vaccines [6], and people’s perception about the seriousness of the disease as a result of the low number of cases and mortality from the disease in Nigeria.

Hesitancy is not the only reason for low vaccine uptake. Vaccine-access and service-delivery bottlenecks also play a role [7]. The WHO developed the Behavioral and Social Drivers (BeSDs) framework and tools in 2018 to help program planners and researchers conduct a comprehensive analysis of the drivers and barriers of vaccine uptake [8]. Using these tools, programs can understand vaccine uptake and hesitancy; track trends over time; and systematically gather data to design, implement, and evaluate tailored interventions to reduce vaccine hesitancy and improve uptake.

The BeSD tool focuses on factors that can be measured in individuals, specific to vaccination, and potentially changeable through programs. The framework is divided into four domains. The thinking-and-feeling domain explains people’s perception of the risk posed by disease and their level of confidence based on perceived safety, trust, and benefits of the vaccine. The social processes refer to social norms relating to vaccination, including health workers’ recommendations. The motivation domain refers to people’s vaccination intention. The practical-issue domain focuses on people’s experience while trying to be vaccinated [8]. Each domain is measured by multiple questions in the full framework and by a single question (priority question) in a pared-down version of the framework. See Table A1 in the Appendix A for domain details. Though different factors drive vaccination, the BeSD framework has been deemed appropriate for use by researchers, program managers, and development partners to understand the reasons and motivations behind people’s intention to vaccinate because it focuses on measurable drivers and can be used to inform decisions on implementation, monitoring, and evaluation.

The BeSD framework has been applied in various studies to understand the drivers of COVID-19 vaccination in different contexts, such as in the United States [9], rural India [7], Malawi [10], and low- and middle-income countries [11], as well as among pregnant women in South Wales [12]. The framework allows for a deeper exploration of context-specific reasons for vaccine uptake. However, as of the time of the study, no published studies have used the BeSD framework to understand COVID-19 vaccination uptake among the general adult population in Nigeria or among adults in a Northern Nigerian state.

To better understand COVID-19 vaccination uptake in Nigeria, we implemented a mixed-methods study to examine the drivers of COVID-19 vaccination among eligible adults in the Gwagwalada Area Council of the FCT, Nigeria, using the BeSD framework. Recognizing the potential cost and time challenges implementing the full range of the BeSD questions may pose to managers in regular programmatic, non-research settings, we also aimed to examine how well the pared-down version of the BeSD items—the priority questions—would perform in predicting vaccine uptake in this setting. This paper addresses two research questions: What are the drivers of COVID-19 vaccination among eligible adults? Can the BeSD priority questions provide the same level of robustness as the multiple questions?

## 2. Materials and Methods

### 2.1. Study Design and Settings

This mixed quantitative and qualitative methods study is part of a larger before-and-after study that tested a community participatory intervention to improve knowledge and uptake of the COVID-19 vaccine among health workers and eligible adults. We implemented this study in the Gwagwalada area council of Abuja, the Federal Capital Territory (FCT) of Nigeria. Gwagwalada is a mixed urban, semi-urban, and rural area council with a population of 475,000. Gwagwalada was chosen because it had the lowest COVID-19 vaccination coverage of the six area councils in the FCT as of January 2022 [13]. The study spanned nine out of the ten wards that make up the Gwagwalada area council. The study did not include one ward because it was considered a security-compromised area. Baseline and end-line evaluation was conducted to ascertain the impact of the intervention.

### 2.2. Participants

The target population for the end-line household survey was individuals aged 18 years and older who were eligible to take the COVID-19 vaccine. The sample size was 1470, estimated based on a background COVID-19 vaccine acceptance rate of 65% in North–Central Nigeria [13], a 95% confidence interval, a 5% margin of error, and 90% power and a 10% non-response rate. We adopted a two-stage sampling technique for the household survey. In the first stage, we divided the local government area into clusters based on the official census enumeration area of the FCT. We then selected 45 clusters using probability proportional to size (PPS) and the appropriate sampling fraction from that sampling frame. In stage two, we enumerated all households in the selected clusters (communities) to create a sampling framework. We then systematically selected 34 households, from which one eligible adult aged 18 years and older was randomly selected to be interviewed. This type of cluster sampling is said to be self-weighting because every unit in the population has the same chance of being selected [14].

For the qualitative data, we purposefully selected the participants based on their characteristics and community roles as follows: being a community leader, religious leader, male and female adults aged between 18 and 49 years, or older male and female adults above the age of 50, and all must have resided in the study area for at least a year. We conducted 4 focus-group discussions (FGDs) and 8 in-depth interviews (IDIs). Each of the FGDs had eight participants. A total of 40 individuals participated in the IDIs and FGDs.

### 2.3. Data Sources and Tools

A structured questionnaire with closed-ended and multiple-choice questions was administered via ODK [15] during the household survey. For the FGDs and IDIs, semi-structured interview guides were used. The tools were developed based on the WHO BeSD framework. They contained domains on demographic information; vaccine behavior; and the four BeSD domains of thinking and feeling, motivation, social processes, and practical issues.

### 2.4. Data-Collection Procedure

The survey was administered using tablets loaded with the questionnaire on the ODK platform to make for easy data collection. Interviewers were expert bilingual data collectors skilled in quantitative data-collection methods. These data collectors were first trained on the study’s data-collection process. We then conducted a pretest of the survey tools in communities that were not part of the original study to check for the tools’ comprehension, logical flow, acceptability, length, and technical quality. Observed errors were corrected, and adjustments were incorporated into the tools. Between 1 and 18 November 2022, the selected research assistants, supervised by the researchers, conducted the household surveys in the study area.

For the qualitative data collection, developed tools were first pretested by the researchers in communities that did not form part of the selected study area. Adjustments to the tools were then made. Qualitative interviews (four FGDs and eight IDIs) were conducted between 1 and 4 November 2022. For each interview, we had an interviewer and a note-taker who took notes on salient points in the interview session. In addition, each interview session was audio-recorded after obtaining the respondent’s permission. Interviews lasted between 20 and 90 min.

### 2.5. Data Analysis

Simple frequencies and proportions were calculated to describe the respondents’ socio-demographic characteristics for all categorical variables. Under the demographic information, age was categorized into three buckets, 18–29, 30–49, and 50+ years; gender was a binary classification of male or female; marital status was defined as never married, married, widowed, or divorced/separated; religion was classed as Islam, Christianity, or traditional; and educational attainment was categorized as none, primary, secondary, graduate, and only Islamic/Arabic education. Islamic education was assigned to those who received no other formal education but had Islamic teachings, typically conducted in Arabic. This category was mutually exclusive from the other educational categories. Employment status was defined as unemployed, self-employed, employed in the private sector, employed by the government, or retired. We also asked about underlying health conditions and provided a list of conditions from which the respondent was asked to choose one condition. Participants were at liberty to select the condition however they deemed fit. For people with multiple conditions, we consider the selected condition the most salient for the respondent.

The mean and standard deviation were presented for continuous variables. We used Pearson chi-square and Fisher’s exact tests to test the association between COVID-19 vaccine uptake and respondents’ characteristics. Socio-demographic characteristics that showed a significant association with a *p*-value less than 0.05 were included in the multiple-variate model.

We built bivariate and multiple-variate logistic regression models to predict the likelihood of getting vaccinated. The outcome variable was vaccine uptake (“Have you received a COVID-19 vaccine?”). This comprises those who are either fully or partially vaccinated. The main predictor variables were drawn from three of the four domains of the WHO BeSD framework. The motivation domain was the fourth domain not included in the models because the question was not asked of those who were already vaccinated. We also note that only one participant reported traditional religion, which caused the models to fail due to “perfect separation”. Since it was not a major category, we removed the participant from the regression models.

Six questions in the thinking-and-feeling domain assessed various aspects of confidence and beliefs about the COVID-19 vaccine, including its benefits, protection, safety, perceived risk of COVID-19, trust in the vaccine, and trust in healthcare workers. Confidence in vaccine benefits was gauged by asking, “How important do you think getting a COVID-19 vaccine will be for your health?” with the ordinal response options of “Not at all important”, “A little important”, “Moderately important”, and “Very important”. It is worth noting that this is the priority question according to the BeSD framework. Perceived protection by the vaccine was assessed with the question “How much do you think getting a COVID-19 vaccine for yourself will protect other people in your community from COVID-19?” with the ordinal responses “Not at all”, “A little”, “Moderately”, and “Very much”. The question “How safe do you think a COVID-19 vaccine will be for you?” was used to assess people’s confidence in the safety of the vaccine, with the ordinal responses “Not at all”, “A little”, “Moderately”, and “Very much”. The question “How concerned are you about getting COVID-19?” was used to assess the perceived COVID-19 risk, with ordinal responses of “Not at all concerned”, “A little concerned”, “Moderately concerned”, or “Very concerned”. Trust in the available COVID-19 vaccine was assessed with the question “How much do you trust the COVID-19 vaccine available for you now? with ordinal responses of “Not at all”, “A little”, “Moderately”, and “Very much”. The question “How much do you trust the healthcare providers who administer COVID-19 vaccine?” was used to assess confidence in healthcare workers, giving the vaccine with ordinal responses “Not at all”, “A little”, “Moderately”, and “Very much”.

Three questions were used in the social-process domain to assess the support of family and friends in receiving the COVID-19 vaccination, the support of community and religious leaders in getting the vaccine, and the willingness of social networks to receive the vaccine. Support of family and friends in getting COVID-19 vaccine was assessed with the question “Do you think most of your close family and friends would want you to get a COVID-19?” with response options of “No”, “Maybe”, and “Yes”. It is worth noting that this is the priority question according to the BeSD framework. Support of community and religious leaders in getting the COVID-19 vaccine was assessed with the question “Do you think your community leaders or religious leaders would want you to get a COVID-19 vaccine?” with response options of “No”, “Maybe”, and “Yes”. Social networks willing to get the COVID-19 vaccine was assessed with the question “Do you think most adults you know will get a COVID-19 vaccine, if it is recommended to them?” with response options of “No”, “Maybe”, and “Yes”.

One question in the practical issue assessed knowledge of where to get vaccinated: “Do you know where to go get a COVID-19 vaccine for yourself?” The response can be either “No” or “Yes”. It is worth nothing that this is the priority question for this domain in the BeSD framework.

Prior to conducting the analyses, we utilized a one-hot encoding to convert the ordinal responses from categorical to numerical for the thinking-and-feeling and social-process domains. As a result, we reported a one-unit change in the perception measure for the thinking-and-feeling and social-process domains, whereas the response for the practical-issue domain was categorical.

In our model building, we first ran bivariate models and regressed the demographic and behavioral variables separately with the outcome. This was to demonstrate how all predictors, especially the different items in the thinking-and-feeling and social-process domains influence vaccine uptake, thus providing an insight into which items will drive the subsequent model that uses a summary score of the behavioral items to predict the outcome.

Then, we ran a multiple-variate model that used the BeSD domain scores to predict the likelihood of getting vaccinated (BeSD composite score model) while controlling for demographic factors. We created a composite index for each BeSD domain by summing their scores from each item response. For example, the thinking-and-feeling domain has six items. On each item, participants can receive a score ranging from one to four. For example, the first item in the domain asks the following question: “How important do you think getting a COVID-19 vaccine will be for your health?” The response options and scores for this item are as follows: “Not at all important” = 1, “A little important” = 2, “Moderately important” = 3, and “Very important” = 4. A similar treatment was performed for the remaining five items. Scores from all six items were then aggregated to obtain a total score for the thinking-and-feeling domain. This summary score was then used in the regression analysis. Higher scores represent more pro-vaccine attitudes.

The logistic regression equation for this model is expressed as follows:

Log (p/1 − p) = β_0_ + β_1_MI Q1 + β_2_MI Q2 + … + β_n_MI Qn

where log (p/1 − p) is the log odds of the outcome; β_0_ is the intercept; β_1_MI Q1, β_2_MI Q2, …, β_n_MI Qn are the coefficients for each predictor variable; and p is the probability of the outcome.

Finally, we ran a second multiple-variate model with demographic control variables, and the BeSD domains represented by only the priority questions. This model predicts the likelihood of getting vaccinated based on the WHO-defined BeSD priority question for each domain. The logistic regression equation for this model can be expressed as follows:

Log (p/1 − p) = β_0_ + β_1_BeSD Q1 + β_2_BeSD Q2 + … + β_n_BeSD Qn

where log (p/1 − p) is the log odds of the outcome; β_0_ is the intercept; β_1_BeSD Q1, β_2_BeSD Q2, …, β_n_BeSD Qn are the coefficients for each predictor variable; and p is the probability of the outcome.

For both multiple-variate models where we had the BeSD composite index and BeSD priority questions as predictors, the outcome variable was vaccine uptake, and the demographic control variables were age, gender, education, and religion. The log odds were exponentiated and reported as odds ratios with their corresponding confidence intervals.

We further compared the composite index and the priority question models to ascertain their fitness and robustness. The McFadden pseudo-R^2^ was used to test each model’s fitness, with values ranging from 0.2 to 0.4, indicating a very good model fit. Also, the Akaike Information Criterion (AIC) model evaluation performance was checked for the two models, with lower AIC models deemed to be more preferred. All quantitative analyses were conducted using the open-source software R (version 4.2.2) in the R-Studio environment.

For the qualitative data analysis, we first checked the quality of the audio recordings from the interviews. We then employed expert bilingual transcribers, who transcribed the English recordings verbatim. The interviews that were conducted in local dialects were transcribed into English. After receiving the transcripts from the transcribers, we performed quality checks on them by back-translating 5% of the translated transcripts to ensure that the concepts were fully carried across. We then independently developed codes along thematic lines for analysis. An initial set of codes was developed deductively based on the BeSD domains and research questions. Subsequently, additional codes were developed inductively during the analysis of the first 10 transcripts to capture emerging themes. The codebook was then harmonized before further coding and analyses were carried out using Dedoose software version 9.0.54, 6 July 2022. Illustrative verbatim quotes are included to support the qualitative insights. In some cases, quotes have been slightly edited for clarity, while retaining the respondent’s voice as much as possible.

## 3. Results

### 3.1. Demographic Distribution of Study Participants

The demographic profile of survey respondents at the end-line and the COVID-19 vaccine uptake by their characteristics are presented in Table 1. Except for age, the characteristics are presented by descending order of their frequencies. The response rate was 100%, with 1512 individuals interviewed. Of these, 65% were female. The mean age was 35 years (SD = 13 years). Respondents were grouped into three age categories: youth (18–29 years), adults (30–49 years), and older adults (50+ years). The largest group was adults (30–49 years), making up 45% of the sample, followed by youth (39%). Most respondents (73%) were married, 64% were Muslim, and 40% had only secondary education. Half were unemployed, and 43% were self-employed. Regarding health status, 91% reported having no underlying health conditions.

In the qualitative piece, a total of 40 respondents were interviewed, of which 81% were FGD participants. Forty-three percent of qualitative respondents were between the ages of 30 and 49, 38% were aged 50 and above, and 18% were between the ages of 18 and 29 years. Fifty-one percent of the respondents were women, 45% were graduates, 29% were secondary-school certificate holders, 13% did not have any formal education, 9% finished primary school, and 5% were postgraduate certificate holders.

Only 46% of the survey respondents have been vaccinated. In the bivariate analysis, the chi-square and Fisher’s exact tests revealed that gender (male; *p* < 0.01), religion (Muslim; *p* < 0.01), and education (graduate and Arabic; *p* = 0.01) were strongly and positively associated with COVID-19 vaccine uptake. Those who had reported having diabetes or being overweight were marginally more likely to be vaccinated than on average—50% versus 46% (*p* < 0.04).

### 3.2. Behavioral and Social Drivers of COVID-19 Vaccine Acceptance

Next, we present the bivariate and multiple-variate logistic regression models to examine the relationship between the BeSD variables and odds of COVID-19 vaccination. Table 2 shows the results of all the models. The separate bivariate regressions shows that all items in the thinking-and-feeling, social-process, and practical-issue domains are independent and statistically significant predators of COVID vaccination. Out of all predictors, the single item in the practical-issue domain—knowing where to get the COVID vaccine—had the largest effect size, (unadj. OR = 4.66, 95% CI: 3.74, 5.81). Within the thinking-and-feeling domain, the item with the strongest association to vaccination was confidence in COVID-19 vaccine safety, unadj. OR = 3.07 (95% CI: 2.67, 3.53); meanwhile, in the social-process domain, the strongest predictor was perceived willingness of one’s social networks to get the COVID-19 vaccine, unadj. OR = 3.16 (95% CI: 2.59, 3.85). Perceived risk of getting the COVID-19 had the weakest association with vaccine uptake, unadj. OR = 1.41 (95% CI: 1.29, 1.54).

In the first multiple-variate model, which examines how COVID vaccination increases as a function of the composite index score from each BeSD domain, we see that one unit increase in the thinking-and-feeling domain score, increases the odds of vaccination by 17% (adjOR = 1.17, 95% CI: 1.13, 1.20; *p* < 0.01). This construct combines various aspects of confidence and beliefs about the COVID-19 vaccine, including its benefits, protection, and safety; perceived risk of COVID-19; and trust in the vaccine and in the healthcare workers administering it. In our qualitative interviews, many participants reported that they were confident in the COVID-19 vaccine available in the country and that the vaccines were effective. Others reported satisfaction with the quality of the vaccine:

*“The vaccine is effective because it works after you take it. People who get vaccinated are getting better, so the vaccine is okay. As long as people are getting it and getting well, it’s good”.*

(IDI with older adult, Central Ward)

*“I will tell them it is good for their health. I have diabetes and I took it, and I am feeling fine. I still need to advise my friend to take the vaccine”.*

(FGD with older adult, Zuba Ward)

*“They should accept the vaccine because it is good. Otherwise, they wouldn’t bring it”.*

(IDI with traditional ruler, Tunga Maje Ward)

Social processes and norms were also important drivers of vaccine uptake. The odds of vaccine uptake increased by 42% (adjOR = 1.42, 95% CI: 1.14, 1.77; *p* < 0.01) for a one-unit increase in the perceived support as captured by the three items in the domain. Participants in the qualitative interviews also reported the influence of family and friends on getting vaccinated. Two participants mentioned their fathers’ role in getting vaccinated. Another mentioned the role the elder sibling played in her being vaccinated. A particular participant mentioned his role in getting his friends and family members vaccinated.

*“Because my family know the importance of the vaccine they ask me to take it”.*

(FGD with young male adult, DOBI Ward)

*“On that day, my father asked me to go take the vaccine. So I stayed back and ate until I was full. When I got there, they asked me for my age and name and wrote it down. I saw people in the queue ahead of me get vaccinated, and then it was my turn, and I got vaccinated too. When I got home, they asked if I felt any pain. I said the pain was minimal, and they told me to take paracetamol for it”.*

(FGD with young female adult, Ibwa Ward)

*“I was in Garki when the pandemic started. When I got home, I asked my father, who is a doctor, about it. He told me that a vaccine was available, so I got vaccinated to avoid contracting the virus”.*

(FGD with young male adult, DOBI Ward)

*“When I heard about the COVID-19 vaccine, I thought about it and decided to get vaccinated. I asked my brother for permission and joined a queue. On getting to the vaccination point, I sat down and waited until it was my turn to get vaccinated”.*

(FGD with young female adult, Ibwa Ward)

*“During that time, people around me were taking the COVID-19 vaccine, so why shouldn’t I? Some of my friends who hadn’t taken it then went ahead and got vaccinated. Even my dad, who is over 70, took it. I informed them all about the vaccine and its side effects”.*

(IDI with young adult, Quarters Ward)

Practical issues like knowledge of where to get the COVID-19 vaccine were also associated with higher vaccination likelihood, as adults who knew where to get the vaccine were more than four times more likely to be vaccinated (adjOR = 4.21, 95% CI: 3.27, 5.43; *p* < 0.01) compared to those who did not. Most of the participants in the qualitative interview reported knowledge of where to get the vaccine, and most also reported that the vaccination sites were close to them. A particular participant reported that the vaccination site being near made it easier for people to get vaccinated.

*“We are a field team, moving from place to place, even to Fulani houses, for COVID-19 outreaches. This helps them get vaccinated”.*

(FGD with young male adult, DOBI)

The second multiple-variate model utilized the priority questions from each domain as the predictor, controlling for demographics. Our findings show that those priority questions are significant predictors of vaccination. The odds ratio associated with the priority questions were slightly higher than shown for a unit change in scores for the corresponding domain, but the different was minimal, suggesting that the priority questions were good proxies for the broader concept represented by a combination of all the items in the domain.

After observing similar findings from both multiple-variate models, we conducted a formal test to compare the two models. The results revealed a pseudo-R^2^ value of 0.25 for both models, falling within the recommended range from 0.2 to 0.4. This indicates that both models provide a good fit for the data. Additionally, the AIC (Akaike Information Criterion) for the composite index model is 1585.3, while for the priority question model, it is 1578.2. The AICs for both models are relatively the same, suggesting that both models demonstrate comparable robustness.

## 4. Discussion

We employed a mixed-methods approach to examine the socio-behavioral drivers of COVID-19 vaccination, using the WHO BeSD framework. Furthermore, we compared the performance of a model that used only one priority question—to assess each domain with a model that utilized a composite score of multiple items per domain. Our study findings demonstrated that all the items in the BeSD domains of thinking and feeling, social processes, and practical issues, emerged as significant drivers of COVID-19 vaccination among the study population.

In the “thinking and feeling” domain, all items were significant predictors of vaccine uptake, with confidence in vaccine safety showing the strongest association in the univariate analysis. This finding was supported by insights from the qualitative data, where participants reported confidence in the effectiveness of the vaccine, as well as satisfaction with the vaccine process. Our results are corroborated by other findings [16] that show confidence as a strong driver of COVID-19 vaccination. This study is part of a larger intervention study that deployed trained, trusted, and influential members of the community to sensitize community members about the COVID-19 vaccine and address their concerns. Confidence in the vaccine could have been boosted by the study interventions, including continuous sensitization and mobilization by trusted community messengers on the safety and efficacy of the vaccines and context-specific messages that focused on individual and community issues about the vaccines.

While we see confidence in the vaccine benefits playing a strong role in its uptake, it is important to acknowledge that the COVID-19 vaccination program in Nigeria has faced challenges due to a lack of confidence and trust in the product and its institutions. This trust deficit is related to rumors, misinformation, and conspiracy theories surrounding the approved vaccines [6]. Such concerns have led to doubts about the efficacy and safety of the vaccines, particularly fueled by skepticism from the perceived rapid COVID-19 vaccine development and deployment timeline compared to historical precedents for other vaccines. People have also questioned the government’s intentions in implementing the vaccination program when it failed to implement a credible palliative program during the lockdown. These and other reasons may account for why 54% of the population was still unvaccinated at the time of our study.

In other settings, these reasons have also been shown to affect vaccination uptake. For example, Tibbels et al. reported a lack of confidence in the vaccines and their development process and distrust in government intentions as drivers of COVID-19 vaccination intention among eligible Ivorian adults [17]. Addressing these concerns, dispelling misinformation, and building trust in the vaccination process are critical steps towards increasing vaccine acceptance and uptake within the population. Effective communication strategies, transparency in providing accurate information, and engaging with communities to address their specific concerns can play a pivotal role in overcoming these barriers and increasing vaccine coverage. Targeted messaging has proven to help dispel the first two factors. Sending context-specific messages that speak to individual concerns about vaccines increases the intention to get vaccinated. Refs. [18,19,20] showed that the adoption of coordinated target-specific messages is a veritable means of improving knowledge and, in so doing, uptake.

We also found that, in the social-process domain, all three items significantly predicted vaccination, with the perceived willingness of one’s social network to get vaccinated showing the strongest association in univariate regression. In multivariate regression, operationalizing the social-process domain either as a composite index of the three items or as the priority question showed the same positive association with vaccination. This finding highlights the significant impact of social support and positive norms on COVID-19 vaccine uptake. The endorsement and support from referent others, particularly family members such as male parents, elder siblings, and friends, emerged as influential factors in the qualitative interviews and focus-group discussions (FGDs) conducted during our study. This aligns with the existing literature that consistently demonstrates the influential role of subjective and injunctive norms in driving behavior change. For example, Antwi-Berko and colleagues conducted a study among minority groups in Amsterdam, Holland, and found that 15.2% of their respondents indicated a greater inclination to receive the COVID-19 vaccine if recommended by their family and friends [21]. Similarly, Xu et al. studied the influence of social networks on COVID-19 vaccine behavior in China and found that health workers who belonged to a social network that communicates COVID-19 vaccination are likely to be less hesitant [22]. Also, a social network analysis of seasonal influenza vaccination by Edge and colleagues found that students’ perceived vaccination coverage amongst their peers correlates with their decision to get vaccinated [23].

These findings underscore the importance of harnessing social networks and interpersonal relationships to promote vaccine acceptance. By actively involving and engaging family members, friends, and other individuals within an individual’s social circle, vaccine programs can capitalize on the influence of these trusted sources to enhance vaccine uptake. Encouraging open dialogue, addressing concerns, and fostering supportive environments can contribute to building positive social norms around COVID-19 vaccination and facilitate behavior change at a community level.

In the practical-issue domain, we found that knowledge of where to get the vaccine is one of the strongest drivers of vaccine uptake, as adults who know where to get the vaccine are four times more likely to be vaccinated. In the interviews conducted, knowledge and proximity of the vaccination centers were reported to promote vaccine uptake. A systematic review on access to vaccination among disadvantaged, isolated, and difficult-to-reach communities in the WHO European Region found that factors such as availability of and proximity to vaccination service was related to higher vaccine uptake [24].

We were keen to understand how the BeSD framework would perform when the domains and items are operationalized in different ways. In the composite index model, when we regressed the BeSD predictors with the scores, the domains were still significant predictors of vaccination uptake, but the odds ratio associated with a unit increase in the domain scores for both the thinking-and-feeling and social-process domains were lower than the odds ratio associated with individual items in bivariate models. This suggests that, while combining items into a single construct retains predictive value, there is an attenuation of effect size when using summary scores as predictors.

We also compared the results from the domain score with the performance of the priority questions and found that both methods effectively captured the relationship between the domains and vaccination. In the thinking-and-feeling domain, the odds ratio for the priority question on the perceived importance of the vaccine to one’s health was more than double that of the composite score from the six items asking about the respondent’s perceived value of the vaccine in protecting others, perception of its safety, concerns about contracting COVID-19, and trust in both the vaccine and the vaccinator. This suggests that the perceived vaccine benefits might be the key factor that motivates individuals to take action by integrating their perceptions of risk, confidence, and trust.

Likewise, in understanding the social processes that influence vaccine decisions, asking only one question on the perceived endorsement by close family and friends for the respondent to get the COVID-19 vaccine performed as well as a three-question index with two additional questions on perceived support of community and religious leaders for the vaccine uptake, and perceived vaccine acceptance by most adults’ respondents know. This shows that the opinions of very close associates, like family members and friends, often encapsulate the social norms that influence people’s decision-making about vaccination. And, lastly, despite positive attitudes and supportive social norms, if there is no knowledge of where to get vaccinated and if access to vaccination is onerous, vaccination uptake will be sub-optimal. A lack of knowledge of the vaccination site will ultimately prevent people from taking up the vaccine.

Our study clearly shows that, in the Nigerian context, the BeSD framework is an effective model for understanding what drives COVID-19 vaccination rates. Furthermore, we demonstrated that using only the priority questions yielded similar predictive-level efficiency as using a composite index to assess domain-specific Behavioral and Social Drivers. The robustness and efficiency achieved using the priority questions in understanding the social and behavioral drivers of vaccine uptake indicate that countries can easily integrate the BeSD priority questions into the different routine data-collection processes, such as coverage surveys, multiple indicator surveys (MICSs), demographic and health surveys (DHSs), the Expanded Programme on Immunization (EPI), and other nationally representative surveys. Currently, the DHS does not collect psychosocial correlates of vaccination behavior, and this is a big gap. A pared-down set of questions, like the priority questions, may increase the likelihood of including the BeSD questions in the DHS. Collecting data on what people think and feel, the social processes that influence them, their motivations, and practical issues that promote or inhibit their intentions to be vaccinated will help in developing more robust evidence-based strategies to increase vaccination uptake and rates.

## 5. Strength and Limitation

A major strength in our study was the use of mixed methods and the concordance between our quantitative and qualitative results, with the qualitative insights providing rich texture and nuance to explain the survey numbers. One limitation to note is that the questions in the motivation domain were not asked to vaccinated respondents since they address the intention to get vaccinated among the unvaccinated or the willingness to recommend the vaccine to others. Because there were no responses on the motivation domain from vaccinated individuals, we could not include it in the models, as it would fail to predict the vaccinated respondents. The BeSD tools focus on individuals’ vaccination-related beliefs and experiences and can be modified through strategic program planning, implementation, monitoring, and evaluation. Sample weights were not applied in the estimation of prevalence; however, our use of PPS—a self-weighting procedure—in the selection of the clusters minimized any bias.

## 6. Conclusions

Our study employed a mixed-methods approach using the WHO BeSD framework to examine the socio-behavioral drivers of COVID-19 vaccination. We compared the performance of a model using priority questions as predictors versus a model using the composite index scores from multiple items per domain. The findings revealed that all BeSD domains—thinking and feeling, social processes, and practical issues—were significant drivers of COVID-19 vaccination. Knowledge of where to get the COVID-19 vaccination, perceived willingness of social networks to get vaccinated, confidence in vaccine safety, confidence in vaccine benefits, and belief in the protective power of the COVID-19 vaccine were the strongest drivers of vaccination. These findings underscore the importance of providing practical information about vaccine schedules and sources, addressing concerns, dispelling misinformation, and building trust to increase vaccine acceptance. This can be done by engaging community leaders, trusted community representatives, religious leaders, and community organizations to help tailor and deliver context and community-specific messages to the communities. We also showed the importance of leveraging social networks as advocates for vaccines. Using the BeSD framework’s priority questions proved efficient in understanding the drivers of vaccine uptake, suggesting that their integration into routine data-collection processes is feasible. Collecting data on people’s thoughts, feelings, social processes, motivations, and practical issues related to vaccination can inform evidence-based strategies to improve vaccine uptake.

## Figures and Tables

**Table 1:** COVID-19 vaccine uptake by respondent characteristics—data from the household survey of eligible adults 18 years and older.

Characteristic	Total n (%)	Vaccinated n (%)	*p*-Value
**Age**			0.06
Mean (SD)	35 (13)		
18–29	590 (39)	252 (43)	
30–49	685 (45)	315 (46)	
50+	237 (16)	123 (52)	
**Gender**			**<0.01**
Female	976 (65)	405 (42)	
Male	536 (35)	285 (53)	
**Marital status**			0.90
Married	1109 (73)	511 (46)	
Never married	350 (23)	157 (45)	
Widowed	44 (3)	19 (43)	
Divorced/separated	9 (1)	3 (33)	
**Religion**			**<0.01**
Islam	964 (64)	503 (52)	
Christianity	547 (36)	186 (34)	
Traditional	1 (0)	1 (100)	
**Education**			**0.01**
Secondary	608 (40)	247 (41)	
Graduate	372 (25)	191 (51)	
None	231 (15)	109 (47)	
Primary	191 (13)	86 (45)	
Only Islamic/Arabic	110 (7)	57 (52)	
**Employment**			0.20
Unemployed	749 (50)	350 (47)	
Self-employed	649 (43)	281 (43)	
Employed in the private sector	51 (3)	27 (53)	
Employed by government	43 (3)	25 (58.1)	
Retired	20 (1)	7 (35)	
**Health condition**			**0.04**
No underlying conditions	1379 (91)	643 (47)	
High blood pressure	62 (4)	17 (27)	
Not sure	25 (2)	11 (44)	
Diabetes	22 (2)	11 (50)	
Other	20 (1)	6 (30)	
Overweight	4 (0)	2 (50)	
**Total Respondents**	**1512**	**690 (46)**	

**Table 2:** Multiple logistic regression of the Behavioral and Social Drivers of COVID-19 vaccine uptake.

Behavioral and Social Drivers of Vaccination	Separate Bivariate Models	Multiple-Variate Model—Composite Index	Multiple-Variate Model—Priority Question
	Unadjusted	Adjusted for Demographics	Adjusted for Demographics
	Odds Ratio (95% CI)	Odds Ratio (95% CI)	Odds Ratio (95% CI)
**Demographics**			
**Age**			
18–29	ref	ref	ref
30–49	1.14 (0.91, 1.43)	1.11 (0.85, 1.46)	1.09 (0.83, 1.43)
50+	1.45 (1.07, 1.96) *	1.56 (1.06, 2.31) *	1.43 (0.97, 2.11)
**Gender**			
Female	ref	ref	ref
Male	1.60 (1.29, 1.98) **	1.16 (0.89, 1.51)	1.28 (0.98, 1.68)
**Education**			
Secondary	ref	ref	ref
Graduate	1.54 (1.19, 2.00) *	1.19 (0.86, 1.63)	1.17 (0.85, 1.62)
None	1.31 (0.96, 1.77)	1.16 (0.79, 1.70)	1.30 (0.88, 1.90)
Primary	1.20 (0.86, 1.66)	1.21 (0.80, 1.82)	1.30 (0.86, 1.96)
Only Islamic/Arabic	1.57 (1.05, 2.36) *	1.11 (0.67, 1.84)	1.19 (0.72, 1.97)
**Religion**			
Christianity	ref	ref	ref
Islam	2.11 (1.70, 2.62) **	2.08 (1.57, 2.75) **	2.06 (1.56, 2.72) **
**Thinking and feeling**		1.17 (1.13, 1.20) **	
Confidence in COVID-19 vaccine benefits	2.93 (2.56, 3.34) **		2.45 (2.10, 2.85) **
Believes a COVID-19 vaccine protects self and others	2.92 (2.55, 3.34) **		
Confidence in COVID-19 vaccine safety	3.07 (2.67, 3.53) **		
Perceived risk of COVID-19	1.41 (1.29, 1.54) **		
Trust in available COVID-19 vaccine	2.70 (2.38, 3.07) **		
Confidence in HCWs administering COVID-19 vaccine	2.70 (2.35, 3.12) **		
**Social processes**		1.25 (1.13, 1.37) **	
Support of family and friends in getting COVID-19 vaccine	2.87 (2.38, 3.46) **		1.42 (1.14, 1.77) *
Support of community and religious leaders in getting COVID-19 vaccine	2.81 (2.30, 3.44) **		
Social networks willing to get COVID-19 vaccine	3.16 (2.59, 3.85) **		
**Practical issues**			
Know where to get COVID-19 vaccine			
No	ref	ref	ref
Yes	4.66 (3.74, 5.81) **	4.21 (3.27, 5.43) **	4.36 (3.38, 5.62) **
Model evaluation			
Akaike Information Criterion (AIC)		1585.3	1578.2
Pseudo-R-squared		0.25	0.25

Note: Boldface indicates statistical significance (** *p* < 0.001 and * *p* < 0.05). CI: confidence interval.

## Data Availability

All the data used to support the results reported in this study are available from the corresponding author upon reasonable request.

## Appendix A

**Table A1:** Showing the BeSD questions.

Domain	Question	Indicator
Thinking and feeling	How important do you think getting a COVID-19 vaccine will be for your health?	Priority
	How much do you think getting a COVID-19 vaccine for yourself will protect other people in your community from COVID-19?	
	How safe do you think a COVID-19 vaccine will be for you?	
	How concerned are you about getting COVID-19?	
	How much do you trust the COVID-19 vaccine available for you now?	
	How much do you trust the healthcare providers who administer COVID-19 vaccine?	
Social process	Do you think most of your close family and friends would want you to get a COVID-19 vaccine?	Priority
	Do you think your community leaders or religious leaders would want you to get a COVID-19 vaccine?	
	Do you think most adults you know will get a COVID-19 vaccine, if it is recommended to them?	
Practical issue	Do you know where to go get a COVID-19 vaccine for yourself?	Priority
